# Private and Shared Taste in Art and Face Appreciation

**DOI:** 10.3389/fnhum.2016.00155

**Published:** 2016-04-13

**Authors:** Helmut Leder, Juergen Goller, Tanya Rigotti, Michael Forster

**Affiliations:** Department of Basic Psychological Research and Research Methods, Faculty of Psychology, University of ViennaVienna, Austria

**Keywords:** art appreciation, facial attractiveness, private and shared taste, beauty, abstract art

## Abstract

Whether beauty is in the eye of the beholder or shared among individuals is a longstanding question in empirical aesthetics. By decomposing the variance structure of data for facial attractiveness, it has been previously shown that beauty evaluations comprise a similar amount of private and shared taste (Hönekopp, 2006). Employing the same methods, we found that, for abstract artworks, components that vary between individuals and relate to personal taste are particularly strong. Moreover, we instructed half of our participants to disregard their own taste and judge stimuli according to the taste of others instead. Ninety-five women rated 100 abstract artworks for liking and 100 faces for attractiveness. We found that the private taste proportion was much higher in abstract artworks, accounting for 75% of taste compared to 40% in the face condition. Abstract artworks were also less affected than faces by the instruction to rate according to others’ taste and therefore less susceptible to incorporation of external beauty standards. Together, our findings support the notion that art—and especially abstract art—crystallizes private taste.

## Introduction

There is increasing awareness that beauty is a powerful aspect of our visual world. Beauty attracts and binds attention (Leder et al., 2010), plays a large role in decisions regarding object design (Hekkert and Leder, 2008), and is pervasive in mating and social interactions (Rhodes, 2006). However, there is still a debate whether the experience of beauty is grounded in certain objective features or whether it is a more subjective process (Leder and Nadal, 2014). If the experience of beauty is driven by objective features, we would expect high agreement between persons and therefore a high amount of shared taste. If it is the result of a subjective, idiosyncratic history of individual experiences, we would expect rather little agreement between persons, and therefore a high amount of private taste. As in many other discussions, neither perspective seems to be entirely true or false. Evidence of common features influencing the beauty of faces (Langlois and Roggman, 1990; Rhodes, 2006), abstract patterns (Jacobsen and Höfel, 2003; Gartus and Leder, 2013), or object designs (Hekkert and Leder, 2008) indicates shared taste to at least some extent. On the other hand, it is also recognized that people have diverging tastes in everyday aesthetic decisions.

If beauty indeed reflects a combination of private and shared taste components, can we put their relative impact in numbers? Hönekopp (2006) introduced an approach where he estimated the relative proportion of private and shared taste by estimating variance components for facial attractiveness judgments (for a more detailed description of his approach, see below). He found that shared and private taste equally contributed to beauty judgments of faces. The evaluation of facial beauty might show a particularly high amount of shared taste because of its evolutionary origin and the pervasiveness of faces in our environment. We are highly familiar with looking at faces and highly sensitive to implicitly and explicitly judging their attractiveness. This experience in judging faces may make people “experts” regarding their own taste. In the arts, and especially in the abstract arts, this is not the case. Even art experts are usually not that pervasively exposed to artworks as we all are exposed to faces. Moreover, the perception and evaluation of facial attractiveness is considered to fulfill biological functions (Little et al., 2011), reflecting several hard-wired mechanisms that contribute to a coherent norm of facial attractiveness (Rhodes, 2006; Swami and Furnham, 2008).

However, regarding the nature of the aesthetic sense, there are many objects for which such an expertise or biologically determined norm can be doubted. For other object categories, the proportions of private and shared components might therefore be different. Especially in the domain of art, individuality and taste are considered particularly important (Leder et al., 2004; Leder and Nadal, 2014). It is a widely held belief that art production and appreciation is not typically governed by universal rules, and consequently aesthetic preferences in the domain of art have been discussed as being highly individual. Leder et al. (2012) argued: “there is hardly any aspect of our everyday perception that seems more subjective than the human appreciation of art. People differ in the type of art that they prefer” (p. 1).

Regarding the coherence of aesthetic evaluations, Vessel et al. (2014) found lower observer agreement for images of artworks and architecture as compared to landscapes and faces. They assumed that preferences for artifacts such as artworks rely on more personalized attributes because of their reduced behavioral relevance and fewer basic-level distinctions (also see Leder et al., 2012). Although it has been argued in art history that abstract art might constitute a universally understandable visual language (Haftmann, 1959), empirical evidence supports the opposite: visual exploration is less coherent and aesthetic evaluations are less similar across observers for abstract as compared to representational art (Brinkmann et al., 2014; Schepman et al., 2015). This difference might be due to more converging associations and more assigned meaning in representational than in abstract art (Schepman et al., 2015). Vessel and Rubin (2010) also argued that for abstract artworks, appreciation might be the result of internal, subjective factors shaped by the viewer’s personal experience. As shared preferences for certain types of artworks have been found to be driven by shared semantic interpretations (Vessel and Rubin, 2010), abstract artworks offer an intriguing research challenge due to their lack of meaningful semantic content, making them objects that can be differentiated only by their style of depiction. Conceptual ideas, stylistic reflections, and variations also are not readily apparent (Leder et al., 2004), making meaning extraction a greater challenge (Belke et al., 2006; Jakesch and Leder, 2009). All this explains the rather low observer agreement in preference, possibly due to this larger need—but also openness—for interpretation. Applying the method of Hönekopp (2006) allows us to add empirical evidence to the debate about private vs. shared taste in evaluating beauty in abstract artworks compared with faces. Thus, we can directly test whether and to what extent private taste drives the aesthetic evaluation in abstract art.

Moreover, we do not simply assume that evaluation of abstract art is marked by a high proportion of private taste. If abstract art represents a class of objects for which private, individual taste is primarily considered, a common standard might not exist. We indirectly tested this by showing that when participants are instructed to disregard or tune out their own taste and acknowledge the taste of others, the effects are weaker for abstract art than for faces. Therefore, apart from the default evaluation based on one’s own taste—“How much do I like it?”—we asked half of our participants to consider the taste of others for their evaluation—“How much do others like it?”. This evaluation, we argue, requires taking the perspective and incorporating the taste of others. Thus, in order to do so, people need to consider a norm regarding what generally is liked or considered attractive (i.e., a beauty standard). For faces, people might be well aware of features that determine such standards, such as smooth skin, youth, or averageness (Langlois and Roggman, 1990; e.g., Fink et al., 2001; Little and Perrett, 2002; Penton-Voak et al., 2003). For abstract artworks on the other hand, such standards might be much weaker, or even nonexistent. When we assume that art evaluations are highly individual, it should be much more difficult to take the perspective of others. We therefore hypothesize that the instruction to evaluate according to the taste of others increases the shared taste proportion for faces, but does so to a lesser extent for abstract artworks.

To measure the contribution of private and shared components of taste, Hönekopp (2006) introduced a method borrowed from generalizability theory (Shavelson and Webb, 1991; Brennan, 2001). By estimating variance components, this method allows testing the suitability of a particular measure for testing a specific effect. Variance components can then be interpreted as observed variance attributed to the measurement objects (tests) and to the facets of the measurement (items, session). This approach can be applied to participants rating any stimuli, such that the participants are the “tests” and the stimuli are the “items”. For decomposing the variance into its components, participants need to rate the attractiveness of a series of stimuli (e.g., faces) twice. The repeated measure is necessary to separate the critical components from the residual. The ratings are then taken to estimate variance components for the rater (also called judge, *varcomp*_J_), the stimulus (*varcomp*_S_), and the judge × stimulus interaction (*varcomp*_J×S_). The more the judges agree, the more variance can be explained by the stimuli and the higher the variance component for a stimulus. Shared taste is therefore reflected in *varcomp*_S_. The variance component for the interaction is an indicator of how much the stimuli scores depend on the judge. Private taste is therefore reflected in *varcomp*_J×S_. The variance component for judges (*varcomp*_J_), however, indicates a general difference between judges. We can either assume that this difference reflects a meaningless difference in scale use, or that it reflects additional variation for private taste. For both assumptions, Hönekopp (2006) provided beholder indices (*bi*_1_ and *bi*_2_) that measure the relative amount of private taste compared to shared taste. Assuming that *varcomp*_J_ is meaningless, *bi*_1_ is computed as *varcomp*_J×S_/(*varcomp*_J×S_ + *varcomp*_S_). Assuming that *varcomp*_J_ reflects meaningful variation, *bi*_2_ is computed as (*varcomp*_J×S_ + *varcomp*_J_)/(*varcomp*_J×S_ + *varcomp*_S_ + *varcomp*_J_).

Following our main hypothesis, for abstract artworks we expect that private taste explains more variance than shared taste. In numerical terms, *bi* should be rather high (or at least above 50%), with the variance component for the judge and artwork interaction explaining the most meaningful variance. For faces—following Hönekopp (2006)—we expect an equal proportion of private and shared taste. When participants are instructed to rate according to the taste of others, for abstract artworks we expect only a small decrease in private taste. For faces, we expect a stronger decrease in private taste, because presumably more common standards are available for rating faces compared to rating abstract artworks.

## Materials and Methods

### Participants

Ninety-five heterosexual (self-reported) women between the ages of 19 and 33 years (*M* = 22.7, *SD* = 2.9) took part in the experiment. In order to keep the experimental design simple, we only tested female participants and presented them only with male faces. Most participants were undergraduate psychology students from the University of Vienna, who fulfilled curriculum requirements or earned extra course credit by participating. All participants had normal or corrected to normal vision (tested prior to the experiment). As art expertise can affect the interpretation and the appreciation of abstract art (Furnham and Walker, 2001; Belke et al., 2006; Leder et al., 2012), we measured art expertise by a self-developed short questionnaire containing knowledge questions about art-facts and artworks; with an average of 34.8% of correct answers (max. 67.6%). Based on our previous use with this scale, we thus considered all participants as non-art experts and included them in our analysis. Prior to the experiment, all participants gave written consent and were informed that participation and data collection were fully anonymous. Participants could withdraw at any time during the experiment without any further consequences. All studies were conducted in accordance with the Declaration of Helsinki (revised, 1983) and the guidelines of the Faculty of Psychology, University of Vienna. We further followed the Austrian Universities Act, 2002 (UG2002)—which was active at the time of the experiments—and which required only medical universities to appoint ethics committees for clinical testing, application of medical methods and applied medical research. Therefore, no additional ethical approval was sought.

### Materials

The estimation of *bi* depends on the distribution of the dependent variable in the sample of images. Hönekopp (2006) argued that in a homogeneous distribution, the impact of private taste could be overestimated. Therefore, we ran pre-studies to select a set of stimuli that showed a relatively heterogeneous distribution in its affective value.

We collected 125 images of abstract paintings from online-databases, such as Prometheus and Saatchi. Images were re-sized so that the longer side equaled 700 pixels, while keeping the original proportions. In a pre-study, 27 participants (17 females) then rated each artwork for liking and valence. Based on the results, images were subsequently split into four groups, combining high/low valence with high/low liking, with 25 artworks selected from each group for use in the main study. Because previous exposure to an artwork has been shown to systematically affect rating (Leder, 2001; Leder et al., 2004), we also ensured in an additional pre-study (*N* = 10) that the selected artworks were largely unknown to laypersons (*M* = 1.43, *SD* = 0.57, measured on a seven-point scale, with one *completely unknown* to seven *well known*).

For the face stimuli, 143 males Caucasian faces were collected from previous studies (*n* = 80, Schacht et al., 2008; Langner et al., 2010), as well as from Google Images (*n* = 63). We edited each image so that the face covered two-thirds of the image in front of a gray background. The age of the faces was in the same range as the age of our participants. Each face showed a relatively neutral expression and was depicted in frontal view, free from occlusions. Where applicable, we used Photoshop (CS2, Version 9.0) to make the additional images from Google similar to the images by Schacht et al. (2008). As in Hönekopp (2006), we scaled all pictures down to a size of 413 × 537 pixels. In a pre-test, 38 females undergraduates rated the attractiveness of all faces. We then split the sample by the median and selected 50 images of each group for the main study. Thus, for both, artworks and faces, we presented 100 images each.

### Procedure

Participants were randomly assigned to an “own taste” or an “others’ taste” group. In the own taste group, participants were instructed to rate the attractiveness of the faces and the liking of the artworks according to their own, individual taste (“It is very important that you rate the pictures/faces based on your PERSONAL TASTE.”). In the others’ taste group, participants were instructed to rate the stimuli as they would be judged by others (“Do NOT rate the pictures/faces based on your personal taste, but based on what you think OTHERS in general would like/find attractive.”).

We presented the artworks and the faces in two subsequent blocks, with the order counterbalanced across participants. The procedure in each block was identical to Hönekopp (2006): in a first series, each artwork/face was randomly presented for 2 s, to allow the participants to form an internal standard for scale use. In a second series, each artwork/face was presented for 5 s and rated on a 7-point Likert-scale. For the artworks, participants were asked “How much do you like the picture?” in the own taste group and “How much is the picture liked in general?” in the others’ taste group. For the faces, participants were asked “How attractive do you find the face?” in the own taste group and “How attractive is the face in general?” in the others’ taste group. A fixation cross was shown for 500 ms between each stimulus during the rating series. One week later, participants returned for a second rating session with the same procedure employed as in the first session.

## Results

### Private vs. Shared Taste

We ran four separate linear random effects models fitted by restricted maximum likelihood (REML) for both groups (own taste and others’ taste) and both stimulus categories (artworks and faces). All analyses were conducted with R (Version 3.1.0, R Development Core Team, 2014) using lme4 (Version 1.1–8, Bates et al., 2015). In addition to the random effects model in R, we ran variance component analysis with SPSS, using either REML or ANOVA Type III as methods (see Hönekopp, 2006). All analyses yielded nearly the same variance components and identical *bi*s. For this reason, we did not report them separately. As in Hönekopp (2006), we included random intercepts for judges, stimuli, time, and all two-way-interactions. Judges represent the participants, stimuli either the artworks or the faces, and time represents the time of testing (first or second session). The variance components and the *bi*s for both groups and stimulus categories are reported in Table 1. Although methods for calculating standard errors and confidence intervals for variance components exist (e.g., Burdick and Graybill, 1992), variance components are usually not normally distributed; thus, “summarizing the precision of a variance component estimate by giving an approximate standard error is woefully inadequate” (Bates, 2010, p. 19). For this reason, we abstained from reporting standard errors or confidence intervals for the estimated variance components. This means that testing for the significance of *bi*s or of the difference between *bi*s is inadequate; therefore, we refrained from doing so.

**Table 1:** **Estimated variance components and beholder indices (*bi*) for artworks and faces in both groups (own and others’ taste)**.

	Artworks		Faces	
	Own taste	Others’ taste	Own taste	Others’ tasteSource of variation
	Estimated variance component (% of total variance)			
Stimulus	0.460 (15.9%)	0.453 (17.9%)	1.179 (44.7%)	1.432 (50.7%)
Judge × Stimulus	1.342 (46.5%)	0.967 (38.2%)	0.677 (25.7%)	0.543 (19.2%)
Judge	0.380 (13.2%)	0.273 (10.8%)	0.260 (9.9%)	0.173 (6.1%)
Session	0.011 (0.4%)	0.005 (0.2%)	0.008 (0.3%)	0.008 (0.3%)
Session × Judge	0.034 (1.2%)	0.049 (1.9%)	0.030 (1.1%)	0.116 (4.1%)
Session × Stimulus	0.006 (0.2%)	0.000 (0.0%)	0.002 (0.1%)	0.000 (0.0%)
Error	0.653 (22.6%)	0.789 (31.1%)	0.480 (18.2%)	0.553 (19.6%)
**Beholder indices *(bi)***
*bi*_1_	0.74	0.68	0.36	0.27
*bi*_2_	0.79	0.73	0.44	0.33

*Note. bi_1_ = beholder index under the assumption that judge-score differences are meaningless. bi_2_ = beholder index under the assumption that judge-score differences are meaningful*.

As indicated in the table, for artworks *bi*s were much higher than for faces. While for artworks, private taste accounted for the major part of the variance (around 73.5% of variance), for faces, shared taste accounted for the major part (around 65% of variance). Moreover, for both stimuli categories, the *bi*s were higher in the own taste group than in the others’ taste group (see bottom of Table 1). As shown in the bottom two rows of Table 1, *bi*_2_ is consistently higher than *bi*_1_. The index *bi*_2_ includes rating differences among participants in the calculation, assuming that this difference adds additional information for private taste. This increase is similar to Hönekopp (2006), but slightly more pronounced for faces than for artworks.

### Group Differences

**Figure 1:**
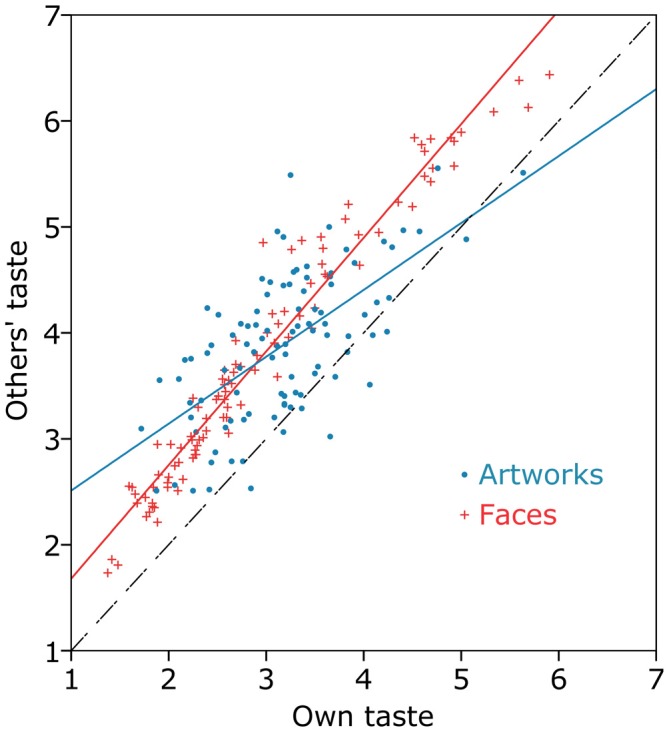
**Relation of the liking/attractiveness ratings between the own taste and the others’ taste group, plotted separately for artworks (blue) and faces (red).** The dashed, black line represents a perfect correlation with no difference between both groups. Dots left of this line represent artworks/faces which have been rated higher in the others’ taste group than in the own taste group. Dots right of this line represent artworks/faces which have been rated higher in the own taste group than in the others’ taste group. The regression lines and the scatter-plots show that both, artworks and faces, were generally rated higher in the others’ taste group. Furthermore, faces show a higher correlation between the groups and therefore a more consistent increase from own taste to others’ taste.

Figure 1 shows the correlation in the ratings between the others’ taste group and the own taste group (shown separately for each artwork, in blue, and for each face, in red). For both faces and artworks, ratings in the others’ taste group were generally higher than ratings in the own taste group (Artworks: *M* = 3.89, *SD* = 0.56; compared to, *M* = 3.18, *SD* = 0.64, *t*_(93)_ = 5.72, *p* < 0.001, Cohen’s *d* = 1.18; Faces: *M* = 3.79, *SD* = 0.49; compared to, *M* = 2.97, *SD* = 0.53, *t*_(93)_ = 7.84, *p* < 0.001, Cohen’s *d* = 1.61). In the figure this is indicated by nearly all dots being to the left of the black dashed line, which represents a perfect correlation. Figure 1 also indicates that faces showed a more homogeneous and systematic increase than artworks. The regression line for faces (in red) is nearly parallel to the black dashed line, whereas the regression line for artworks (in blue) is shallower. This means that for each single face similar differences between own and others’ taste were found. However, some artworks showed no change or even an opposite pattern. This difference between both object categories is also reflected in a higher correlation between the own taste and the others’ taste group for faces (*r* = 0.98, *p* < 0.001) than for artworks (*r* = 0.64, *p* < 0.001).

### Reliability

Cronbach’s alpha is often used as an indicator for the reliability of ratings, with high indicating strong agreement and a high amount of shared taste. However, because reliability is a function of the sample size, even a very moderate agreement among judges can lead to high alphas (Hönekopp, 2006). Thus, Cronbach’s alpha may strongly overestimate the agreement and poorly reflects the actual amount of shared taste. Nonetheless, for the sake of completeness, Cronbach’s alphas, inter-judge agreement, and re-test reliability were also calculated for the data. As expected, for both groups and for both sessions, the reliability of the stimuli scores for artworks was Cronbach’s αs = 0.92, and for faces, Cronbach’s *αs* > 0.98. The average inter-judge agreement for faces was, *r*s = 0.48–0.58, and for artworks, *r*s = 0.19–0.21. The re-test reliability was, on average, *r* = 0.80, for faces and, *r* = 0.71, for artworks.

## Discussion

In the present study, we disentangled private from shared proportions of taste when people judged abstract artworks for liking and faces for attractiveness. Additionally, we tested whether participants—when instructed to do so—can detach their own taste for a stimulus and evaluate the taste of others instead. Our analyses show two main results: first, proportions of private taste were much higher in abstract artworks than in faces, accounting for about 75% of taste in abstract artworks compared to 40% in faces. Second, as expected, abstract artworks were less affected by the instruction to rate the taste of others than faces and therefore less susceptible to common beauty standards.

In line with our main hypothesis, private taste was considerably higher for abstract artworks, explaining around 75% of the total variance (*bi*_1_ = 0.74 and *bi*_2_ = 0.79). This finding is contrary to the art historical belief that abstract art, due to a lack of semantic content, is an universal expression of pure emotion, experienced similarly across different perceivers (see also Brinkmann et al., 2014). It seems, to the contrary, that abstract art is not a universally understandable, or coherently understood visual language (Haftmann, 1959). Even when confronted with abstract combinations of certain colors or shapes—for which the preference should be largely shared if these are truly aesthetic primitives (see Palmer et al., 2013, for an overview)—individuals differ in their evaluations. Even though the presented paintings showed no discernible objects, where individuals might differ in whether they like them or not, abstract artworks still offer opportunities for subjective interpretation. It is possible, that abstraction and lack of semantics simply do not lead to similar experiences and thus to similar preferences. They rather invite individuals to relate the abstract content to vastly different, personal interpretations, and thus to different experiences and preferences. In other words, a depiction of a landscape might be liked by one participant, but disliked by another, but in both cases, experiences about landscapes are triggered. However, a Jackson Pollock painting might remind one participant about his/her untidy desk, whereas it reminds another about his/her untidy lifestyle. Thus, abstract art might invite divergent interpretations (Leder et al., 2004).

In faces on the other hand, the proportion of private taste was much smaller, accounting for only about 40% of the meaningful variance (*bi*_1_ = 0.36, *bi*_2_ = 0.44). Those values are comparable to previous results for women judging men (*bi*_1_ = 0.37, *bi*_2_ = 0.53; Hönekopp, 2006, p. 205).

We further addressed the question of whether the proportion of shared taste can be increased by explicitly instructing half of our participants to evaluate the stimuli according to how they think others would judge them. Thereby, shared taste increased for both object categories, but is was more pronounced for faces (around 10%) as compared to abstract artworks (6%). On first glance, the additional 6% suggest that participants somehow managed to find at least some beauty standard for abstract artworks. However, the artworks themselves only explained an additional 2% of variance, while the remaining variance gain was due to a much higher error component than in the own taste group. We therefore argue that the others’ taste instruction did not considerably influence evaluations. The findings rather indicate that participants have a weak representation of the taste of others for abstract artworks. This can be interpreted in at least two ways: first, the liking of abstract art simply may not have a great amount of shared components, thus these components in others also cannot be inferred. This is also indicated by the low amount of shared taste that we found for abstract artworks (around 25%). The lack of such standards consequently leaves room for stronger private taste, for example personal dislike of certain colors or different associations as argued above. Second, perhaps our participants were not able to infer the shared components, due to the lack of expertise, or experience in knowing about other peoples’ taste for art. In the domain of facial beauty, standards might be much more obvious through media and interpersonal exchange on the topic. The role of expertise could be addressed by testing art experts on abstract art in a similar study design. However, in the study by Brinkmann et al. (2014), when the similarity of gaze patterns was tested, art expertise did not influence the findings.

Regarding facial attractiveness, the decrease of private taste in the shared taste group was more substantial, even though the shared component was already at a higher level. The variance component of the face explained an additional 6% in the others’ taste group as compared to the own taste group. Thus, participants apparently adopted a beauty standard in judging facial attractiveness: they have a certain representation about the taste of others. This finding therefore suggests that our sense of beauty can be part of our understanding of a theory of mind (Wimmer and Perner, 1983).

Interestingly, for both object categories we found that when taking the perspective of others, participants also systematically gave higher ratings than participants in the own taste group. Thus, they were less inclined to respond that others disliked an abstract artwork or face. This effect might be grounded in the participants’ concepts of the others. By rating the taste of others, they might have referred to a social group (out-group) different from their own peer group (undergraduate students). This could have resulted in false uniqueness. That is, individuals may have believed themselves to be more individual (in our case more critical) than the norm (Hoorens, 1993). Additionally, this effect might be explained by a selective misattribution. Participants might have attributed perceptual features of abstract artworks or faces, and which they did not like, to their private taste, while they attributed features which they did like to shared taste. Moreover, abstract artworks showed a less systematic increase than faces when taking the perspective of others. The more systematic increase in faces might be an indicator of strong representations of beauty standards for faces. In abstract artworks however, the lower correlation and the less systematic pattern hint at much weaker standards of what is commonly seen as beautiful.

Despite the clear difference between the amount of private and shared taste between abstract artworks and faces, the findings are not without limitations. First, up to now, there have been unfortunately no meaningful statistical method available to compute whether the differences in beholder indices between both object categories are significant or not (Bates, 2010). Thus, it is also not fully clear whether the differences between the own and others’ taste groups are substantial or within the margins of measurement error. However, given that we could replicate the findings of Hönekopp (2006) with faces, we are confident that the proportions of private and shared taste represent a stable effect. Nonetheless, we hope for future developments in statistical modeling that allow us to corroborate our findings.

By using only unknown faces/artworks, we do not know how famous artworks or faces would affect the proportion of private and shared taste. It seems plausible that the shared taste component would be higher for famous stimuli. However, in faces, factors such as fame or infamy, which might lead to well-known faces but evoke differing responses tied to individual’s own ideas about these concepts, might render the relationship more complex that it seems at first glance. This would be an interesting avenue for further research.

Furthermore, the results of our study are limited to female participants and we did not include any personality traits, which could have influenced the results. For example, for artworks, art expertise might have an effect (Augustin and Leder, 2006; Leder et al., 2012). For faces, factors like sociosexual orientation or relationship status might play a role (Simpson and Gangestad, 1991), in that people with high mate-search motivation not only might assign higher values of attractiveness, but might also show more individual patterns and thus lower the level of coherence. Nonetheless, given the complexity of the design when additional interactions between the gender of the participant and the gender of the stimulus face are tested, and given the weak impact of gender in the previous study by Hönekopp (2006), we opted for only testing female participants. Furthermore, to our knowledge, for abstract artworks there is no strong indication that women differ from men in their preference.

To sum up, we disentangled private from shared proportions in preference for abstract artworks and faces. The private proportion was much higher in abstract artworks than in faces, accounting for about 75% of taste compared to 40%. For the first time, we can add concrete and reliable numbers to the debate of private vs. shared taste in art perception. We also found that instructing the participants to rate the images according to the taste of others increased shared taste for faces, but less so for abstract artworks. These findings are in accordance with the assumption that beauty standards exist for faces, but much less so for abstract art. Thus, preference for abstract art is indeed in the eye of the beholder.

## Author Contributions

All authors designed the study and have written the article together. Study was conducted by TR, and analyzed by TR, JG and MF.

## Funding

This article was supported by grants from FWF (Austrian Science Fund, project numbers P23538 and P27355) to HL.

## Conflict of Interest Statement

The authors declare that the research was conducted in the absence of any commercial or financial relationships that could be construed as a potential conflict of interest.
